# Esthetic orthodontic management with 3D-printed customized brackets for a Class II malocclusion with severe arch-length discrepancy: A case report

**DOI:** 10.1097/MD.0000000000046057

**Published:** 2026-05-12

**Authors:** Viet Anh Nguyen, Thi Minh Anh Ha

**Affiliations:** a Faculty of Dentistry, Phenikaa University, Hanoi, Vietnam; b Viet Anh Orthodontic Clinic, Hanoi, Vietnam.

**Keywords:** angle Class II malocclusions, facial asymmetry, orthodontic anchorage techniques, orthodontic appliances, personalized medicine, tooth crowding

## Abstract

**Rationale:**

Customized lingual appliances fabricated using 3D printing technology offer precise tooth movement and address aesthetic concerns. This case report presents the successful treatment of an adult patient with a Class II malocclusion using this innovative approach.

**Patient concerns:**

A 22-year-old female presented with chief concerns about crowding and a desire for discreet orthodontic treatment.

**Diagnosis:**

Clinical and radiographic examinations revealed a Class II malocclusion with moderate crowding, increased overjet, and a hyperdivergent facial pattern.

**Interventions:**

A comprehensive treatment plan was developed utilizing customized 3D-printed lingual appliances in conjunction with premolar extractions, mini-implant anchorage, and intermaxillary elastics.

**Outcomes:**

After 25 months of active treatment, the patient achieved a Class I occlusion with ideal intercuspation, improved facial profile, and resolution of crowding. The treatment outcome remained stable at a 1-year follow-up appointment.

**Lessons:**

This case report demonstrates the effectiveness of customized 3D-printed lingual appliances in achieving predictable and efficient orthodontic treatment while addressing patient aesthetic concerns. Advancements in 3D printing technology promise to further enhance the precision, efficiency, and accessibility of this approach in the future.

## 1. Introduction

In recent years, the demand for aesthetic orthodontic treatment among adult patients has steadily increased. Often, these patients seek alternatives to conventional metal braces, opting for more aesthetically pleasing options such as Invisalign, ceramic braces, and lingual appliances. Previous studies have shown that Invisalign may not be as effective as fixed appliances in treating complex malocclusions involving tooth extractions. This is due to issues such as tipping into the extraction space and poorer torque control.^[1]^ While ceramic braces are less noticeable than traditional metal braces, the metal archwire can still be visible when smiling. Consequently, lingual appliances have emerged as the preferred choice due to their high aesthetic appeal and proven effectiveness in orthodontic treatment. However, this technique is generally challenging for general dentists and even orthodontists due to its unique biomechanical approach compared with conventional labial orthodontics.^[2]^ Customized brackets can significantly reduce difficulties in lingual orthodontic treatments; however, they incur high laboratory costs.

The increase in orthodontic software and free 3D design software has enabled orthodontists and dental technicians to overcome the obstacles inherent in customized appliance design and fabrication. By digitally designing and creating customized lingual brackets and archwires, production time can be reduced, bracket modification for combining with other specialized appliances is enabled, and associated laboratory charges are lowered.^[3]^ As a result, customized lingual orthodontic treatment has become a more cost-effective and flexible option.^[4]^

Arch-length discrepancies, often manifesting as crowding, are a major concern for patients seeking orthodontic intervention.^[5]^ Severe discrepancies exceeding 10 mm often necessitate bicuspid extraction to prevent excessive labial tipping of the anterior teeth.^[6]^ However, customized bracket design in these cases can be challenging due to limited access to the lingual tooth surface, particularly for severely displaced teeth, hindering accurate digital impressions. Adequate space creation facilitates the use of 3D software to design and fabricate customized brackets via 3D printing. This approach offers enhanced precision and personalized fit, resulting in more accurate bracket placement compared with regular brackets and increasing the versatility of the digital workflow.^[7]^

This study presents a clinical case of a patient with Class II occlusal relationship, severe crowding, and mild protrusion who was successfully treated using a fully customized lingual orthodontic approach combined with bicuspid extraction and mini-implant anchorage. The case highlights the efficacy of customized lingual brackets in achieving optimal treatment outcomes.

## 2. Case presentation

### 2.1. Diagnosis and etiology

The female patient was 22 years old presented with chief complaints of a strongly anterior dentition crowding and slight protrusion. Her medical, family, and psychosocial histories were unremarkable. Regarding dental histories, she had previously undergone endodontic treatment on the mandibular right first molar and had a poor porcelain crown with a chipped occlusal surface and exposed margin.

**Figure 1.**
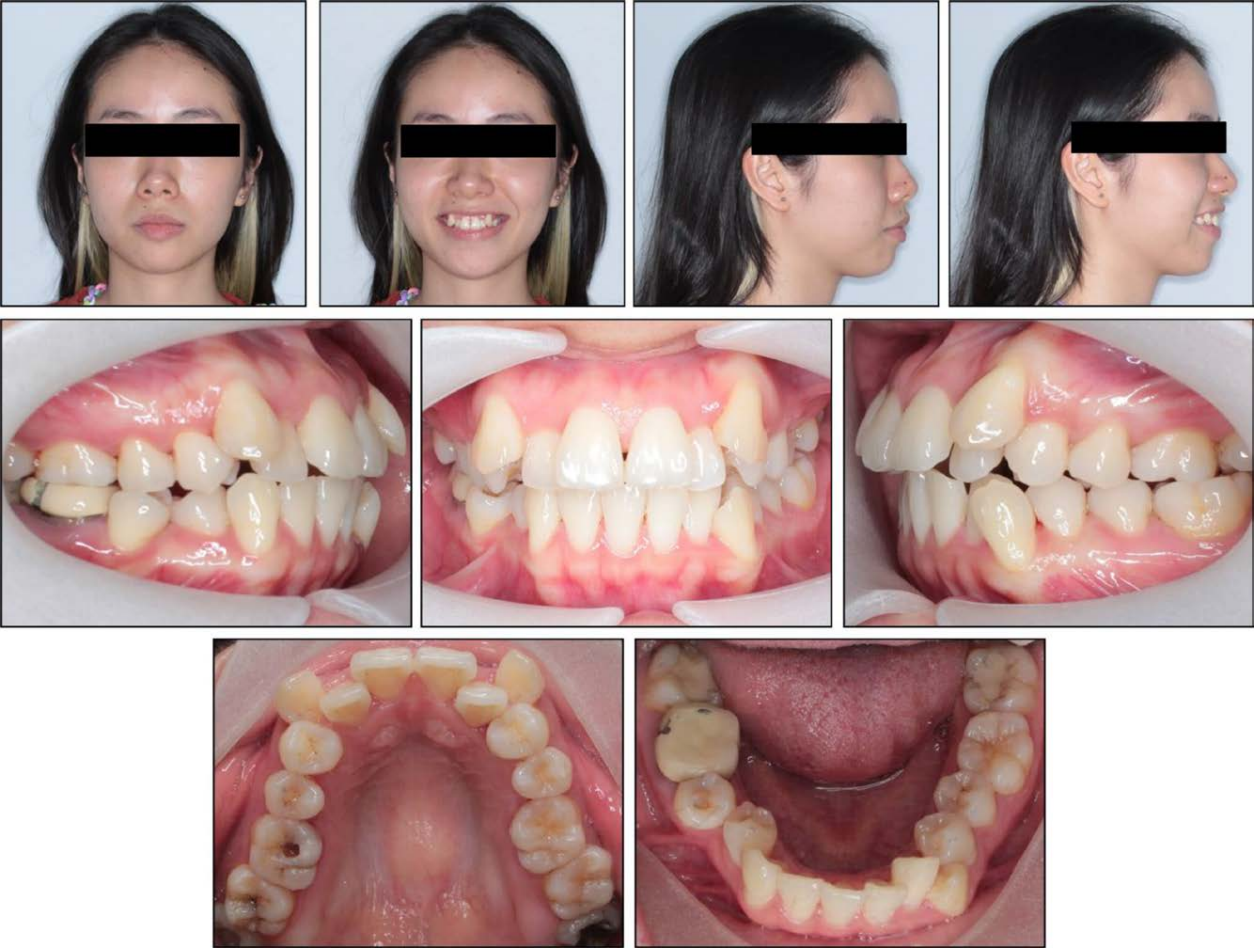
Pretreatment extraoral and intraoral photographs.

Extraoral examination revealed well-proportioned facial thirds and a mild facial asymmetry, with the chin shifted to the left (Fig. 1). The profile analysis showed a convex profile characterized by excessive lip protrusion and lip strain at rest. While a significant 1.8-mm discrepancy was observed among centric relation and intercuspal position, no clinical signs or symptoms indicative of temporomandibular joint disorders were present.

Intraoral examination revealed Class II molar and canine classifications, with a right end-on and left full-cusp occlusion. Overbite was within normal limits at ~1 mm, while overjet measured 3 mm. The maxilla presented a 16-mm space deficiency, and the mandible had a 7.5-mm deficiency. The maxillary midline was shifted 2 mm to the left relative to the facial midline, while the mandibular midline deviated 5 mm to the left. Both maxillary canines were labially positioned. The mandibular incisors exhibited moderate crowding. Additionally, the maxillary left canine and mandibular right first bicuspid were positioned buccally to the mandibular arch. Significant dental caries was noted on the occlusal surfaces of the molars (Fig. 1).

A craniofacial analysis confirmed a mild skeletal Class II malocclusion with a point A-nasion-point B angle of 4.43° (Table 1). The patient exhibited a slightly hyperdivergent facial pattern with an increased Frankfort mandibular plane angle of 31.50°. The maxillary incisors were normally inclined, while the mandibular incisors were proclined, as evidenced by upper incisor to sella-nasion and lower incisor to mandibular plane angles of 102.75° and 103.19°, respectively. Soft tissue analysis revealed a convex profile with the lower lip positioned 3.76-mm anterior to the E-plane. The upper lip was normally positioned at 1.96-mm anterior to the E-plane.

**Table 1:** Cephalometric measurements.

	Pretreatment	Posttreatment
Skeletal		
SNA (°)	77.75	77.02
SNB (°)	73.32	72.30
ANB (°)	4.43	4.72
FMA (°)	31.50	31.15
Dental		
U1-SN (°)	102.75	98.88
U1-NA (°)	25	21.26
U1-NA (mm)	5.97	3.18
L1-MP (°)	103.19	97.84
L1-NB (°)	35.44	29.26
L1-NB (mm)	9.14	6.99
Interincisal angle (°)	115.13	124.86
Upper incisal display	2.03	
Soft tissue		
Upper lip/E-line (mm)	1.96	0.52
Lower lip/E-line (mm)	3.76	1.60

ANB = point A-nasion-point B, FMA = Frankfort mandibular plane angle, L1-MP = lower incisor to mandibular plane, L1-NB = lower incisor to nasion-point B, SNA = sella-nasion-point A, SNB = sella-nasion-point B, U1-NA = upper incisor to nasion-point A, U1-SN = upper incisor to sella-nasion.

**Figure 2.**
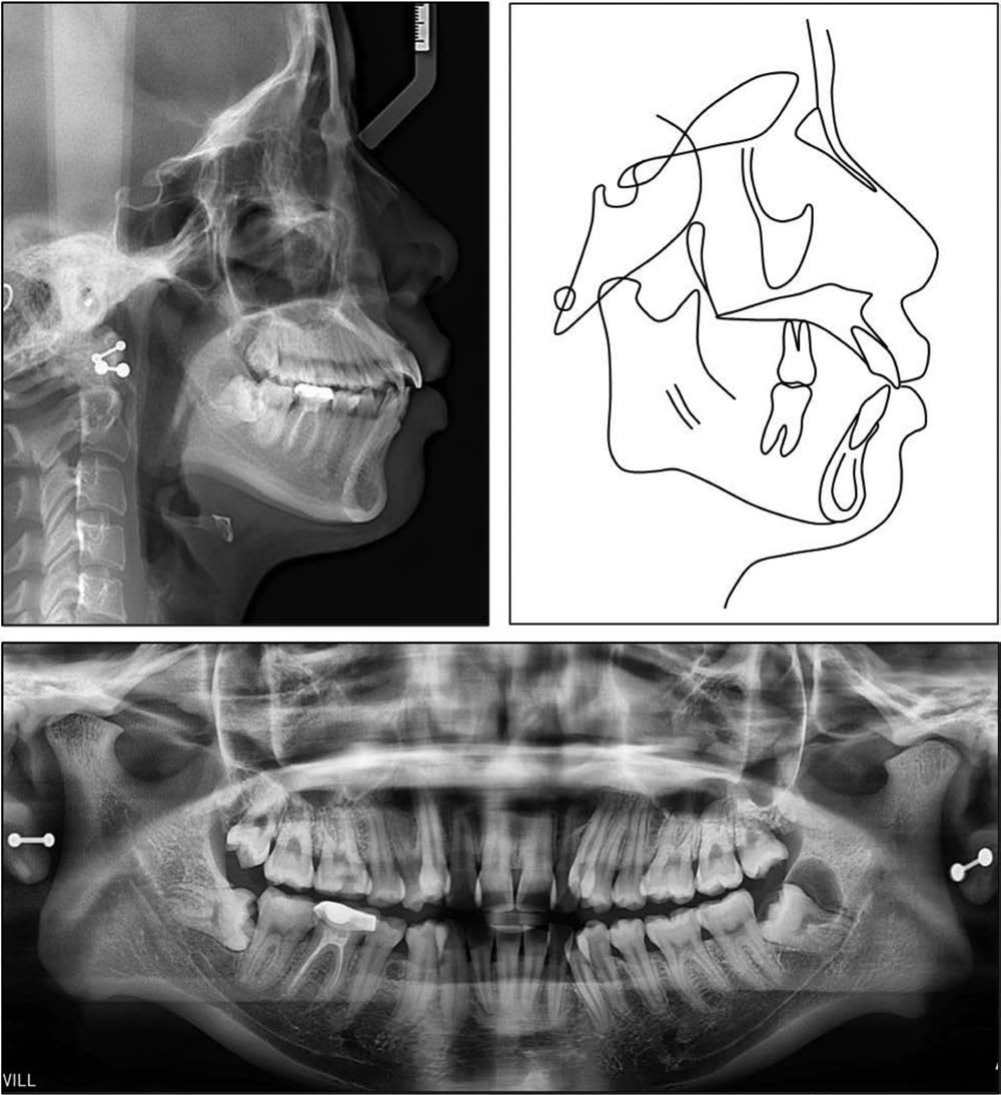
Pretreatment cephalometric and panoramic radiographs and tracing.

Panoramic radiographs revealed impacted mandibular third molars, with a radiolucency associated with the left third molar. Overlapping of the anterior teeth was noted on periapical radiographs of both arches. The mandibular condyles appeared shorter than average bilaterally (Fig. 2).

### 2.2. Treatment objectives

Based on the diagnosis, the primary treatment goal is to enhance the patient’s smile esthetics through comprehensive orthodontic treatment. This includes aligning the severely malposed teeth in both arches, correcting the Class II molar and canine relationship bilaterally, maintaining the maxillary anterior teeth’s inclination while uprighting the mandibular anterior teeth, and achieving a balanced facial profile with proper midline alignment.

### 2.3. Treatment alternatives

Given the patient’s aesthetic concerns, lingual appliances were selected over traditional labial brackets. Since the patient has a mild skeletal Class II relationship, a nonsurgical treatment plan was chosen. To address the significant space deficiency, increased overjet, and hyperdivergent facial pattern, an extraction treatment plan was selected. This plan incorporated absolute maxillary posterior anchorage with skeletal mini-implant support and reciprocal mandibular anchorage. Non-extraction treatment plans often exacerbate the vertical dimension in such cases, potentially leading to a more open bite.^[8,9]^ To address the patient’s Class II malocclusion and facilitate the mesialization of the mandibular anterior teeth, extraction of the maxillary first premolars and mandibular second premolars was indicated. Additionally, due to the impacted mandibular third molars and associated cyst, extraction of all four third molars was included in the treatment plan.

### 2.4. Treatment progress

Following the initial examination, all carious lesions were treated, and the deficient crown on the mandibular right first molar was replaced. As planned, a maxillofacial surgeon extracted the premolars and third molars and surgically removed the associated cyst. Subsequent pathological analysis confirmed the presence of an inflammatory collateral cyst.

**Figure 3.**
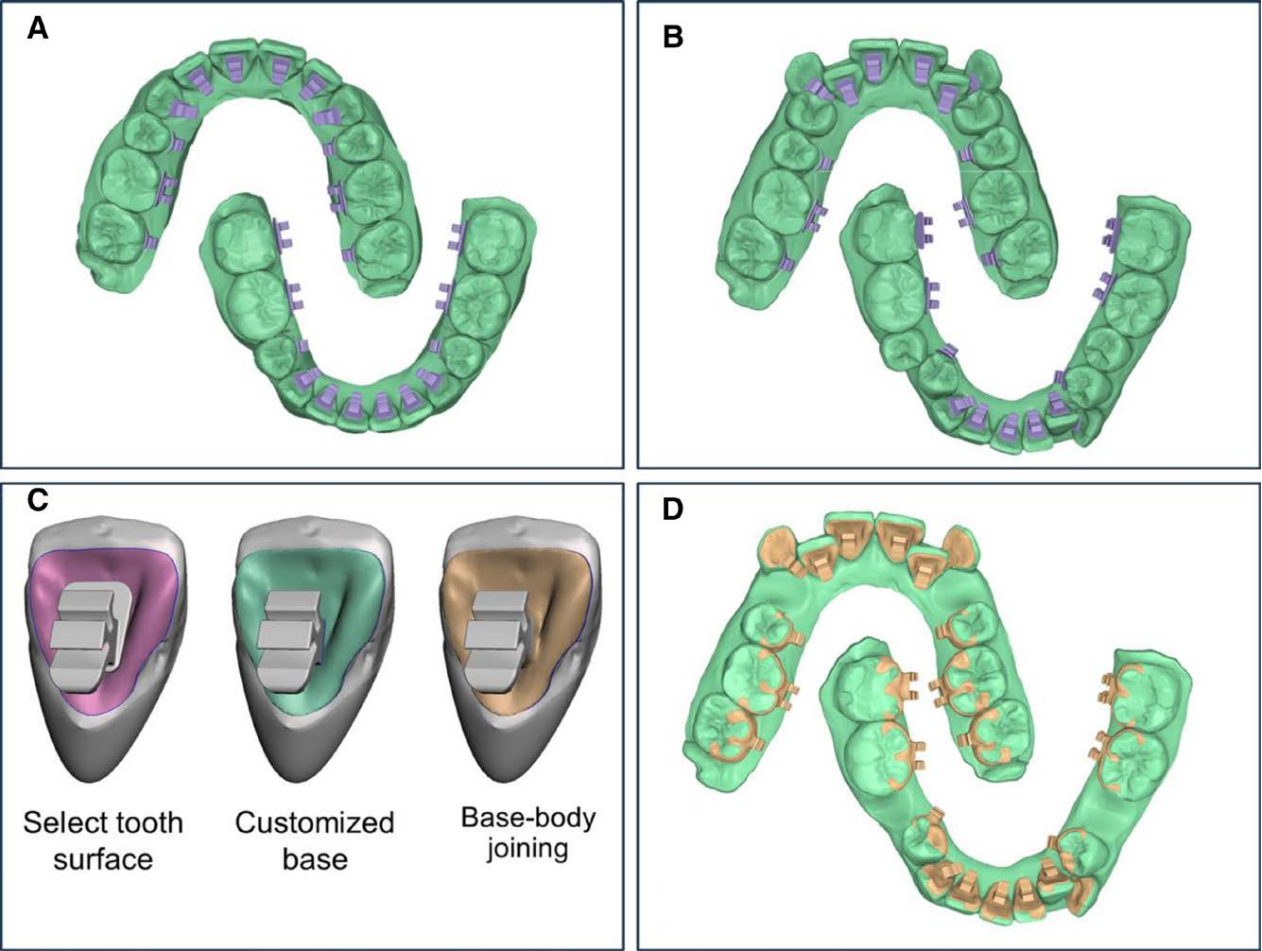
Workflow for customized lingual bracket design: (A) library lingual bracket patches positioned on the orthodontic setup along customized archwires; (B) tooth–patch combinations returned to the pretreatment crowding; (C) per-unit base design in open-source software; (D) completed customized bracket set for metal printing and indirect bonding template fabrication.

Initially, the patient’s dental data were collected with an intraoral scanner and processed using orthodontic software (Autolign, Diorco, Gyeonggi-do, South Korea). This process involved segmenting the teeth and gingiva, constructing a setup model with planned final tooth positions, and establishing virtual arch forms. Predesigned 0.018 × 0.025-inch lingual bracket patches were extracted from the software library and positioned close to the lingual tooth surface along virtual customized archwires. To ensure that prescription would not be constrained by stock bases, the predesigned 0.018 × 0.025-inch lingual bracket patches were used only for slot geometry and tie-wing morphology; the original stock bases were removed later and replaced by a fully customized base. Because brackets were positioned on a setup in which the main slots were aligned to a smooth virtual curve and leveled on the same plane, any mainstream prescription would be acceptable; in this case, we specified zero tip (angulation) for all teeth, and torque values of +55° upper incisors, +45° lower incisors, +5° upper premolars, 0° lower premolars, +10° upper molars, and −10° lower molars to facilitate rectangular wire engagement and torque expression during finishing. Before base design, each tooth–patch combination was returned from the setup to its initial crowded position to keep the base intimately matched to the real pretreatment surface. These tooth-bracket patch combinations were then exported to Meshmixer (Autodesk, California), a free and open-source software, to design customized lingual bracket bases and complete the customized appliances (Fig. 3A–D). The customized base was created by selecting the lingual area intended for bracket placement, smoothing the boundary, offsetting the patch 0.03 mm from the tooth surface to create an adhesive gap, and extruding 0.30 mm to obtain a rigid, printable base. The stock base on each bracket patch was deleted (body retained), and the customized base was boolean-joined to the bracket body to complete each customized unit.

After finalizing the appliance design, the customized brackets were 3D printed using a laser powder bed fusion process with a cobalt–chromium alloy on an NCL-M1100 metal 3D printer (Chamlion, Nanjing, China). The medical-grade cobalt–chromium alloy conforming to ISO 22674 Type 5 criteria for fixed dental prostheses; exhibiting a 0.2% proof stress >500 megapascals, ultimate tensile strength of ~900 to 1100 MPa, elongation at fracture between 2% and 10%, a Young modulus of about 200 to 230 GPa, and a Vickers hardness between 350 and 450, with excellent corrosion resistance consistent with dental standards.^[10–12]^ To maximize slot fidelity, parts were built at 50-µm layer thickness with each bracket oriented at 90° so that the mesial or distal face pointed downward for support attachment, thereby avoiding supports in the slot while maintaining adequate heat dissipation and feature stability during scanning.^[13,14]^ Virtual models combining pretreatment data with the designed brackets were also 3D printed, this time using a Photon D2 digital light processing printer (Anycubic, Shenzen, China), to create thermoformed indirect bonding templates. These templates were fabricated with soft BIOPLAST splints (Scheu, Iserlohn, Germany) using an AX-PMU4 vacuum pressure molding unit (IRIS, Tianjin, China).

After fabricating the customized lingual brackets and indirect bonding trays, the brackets were bonded to the lingual surfaces of the teeth using Single Bond adhesive (3M, Illinois) and GoTo light-cured bracket adhesive (Reliance, Illinois). Orthodontic treatment commenced with initial flexible nickel–titanium 0.012- and 0.014-inch archwires. The archwire sequence then progressed to stiffer nickel–titanium archwires, including 0.016 and 0.016 × 0.022 inch.

**Figure 4.**
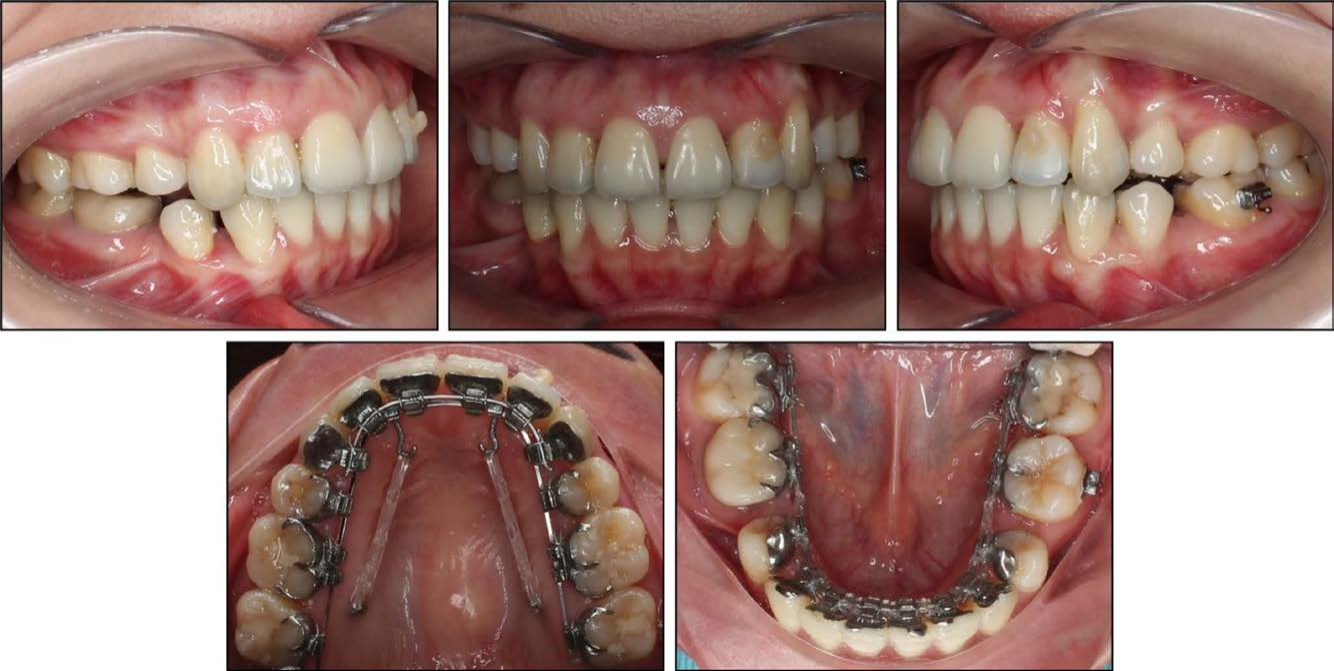
Space closure with skeletal anchorage in the maxillary arch and reciprocal anchorage in the mandibular arch.

After a 6-month leveling phase, excessive anterior protrusion was observed. To initiate space closure, 0.016 × 0.022-inch stainless steel archwires were engaged, and two 1.6 × 10-mm palatal Hifix mini-implants (Medico, Gyeonggi-do, South Korea) were placed for posterior anchorage reinforcement (Fig. 4). Power hooks and elastomeric chains were applied to retract the protruding maxillary anterior teeth. In the mandibular arch, reciprocal space closure mechanics were utilized with elastomeric chains. Class II elastics (3/16 inch, 3.5 oz) were applied on the left side to correct the full-cusp Class II relationship and midline shift.

After 6 months of space closure, 0.017 × 0.025-inch stainless steel archwires were engaged to achieve full expression of the bracket prescriptions. Following 10 months of traction, a Class I dental relationship was achieved on the right side, while the left side remained Class II. To maintain the Class I relationship on the right side and correct the mandibular dental midline deviation, an additional 2.0 × 12-mm buccal shelf mini-implant was placed on the right side of the mandible. After 25 months of orthodontic treatment, the appliances were removed following midline correction and the achievement of a stable occlusion. A lower lingual retainer was bonded to the 6 mandibular incisors, and an Essix retainer was provided for the maxillary arch to maintain long-term stability.

### 2.5. Treatment result

**Figure 5.**
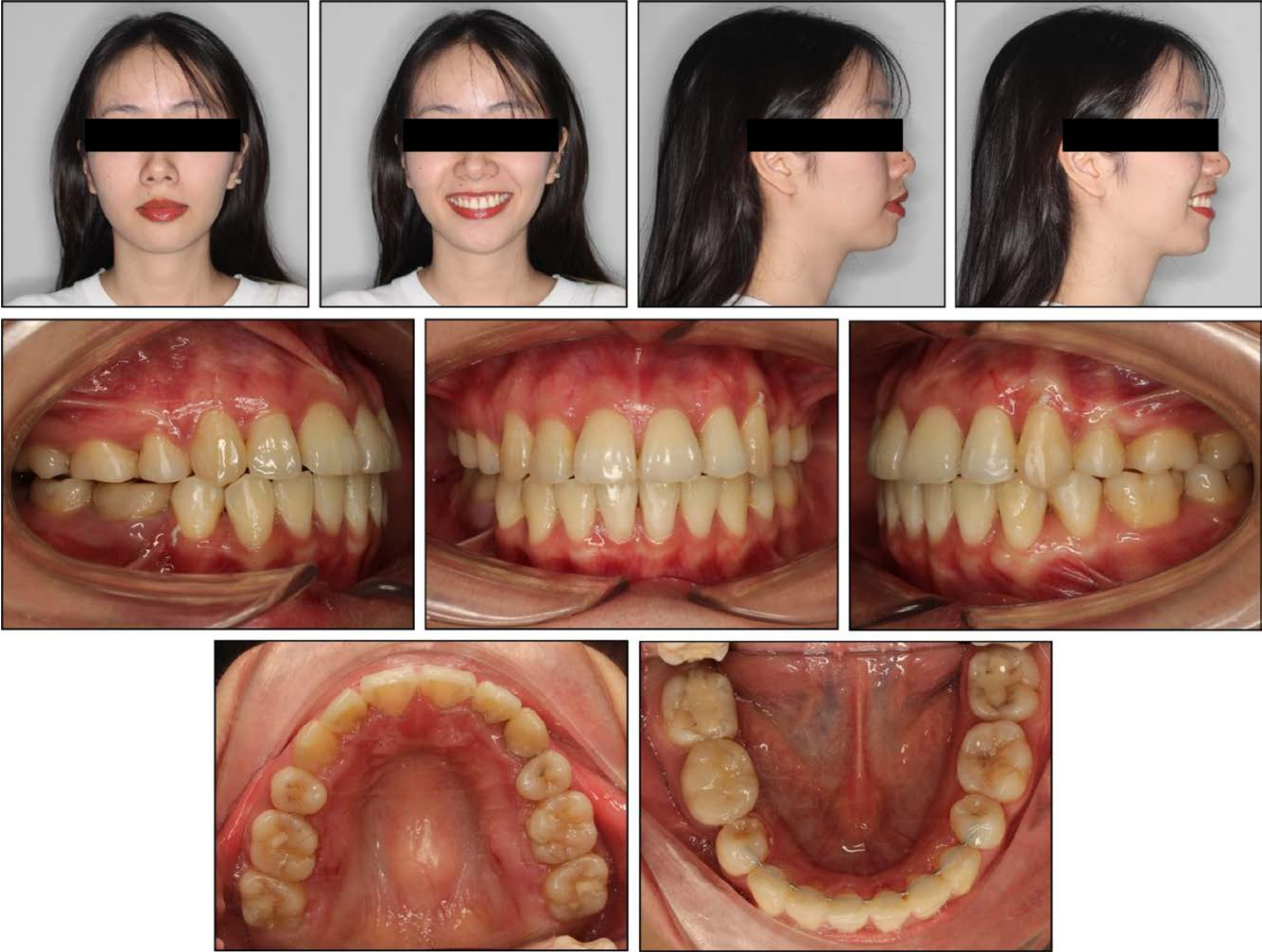
Posttreatment extraoral and intraoral photographs.

The orthodontic treatment successfully achieved the patient’s aesthetic and functional expectations. Treatment goals were effectively met, including correction of the Class II malocclusion to a Class I occlusion through strategic anchorage planning, alignment of the displaced teeth, establishment of centered midlines, and reduction of mouth protrusion (Fig. 5).

**Figure 6.**
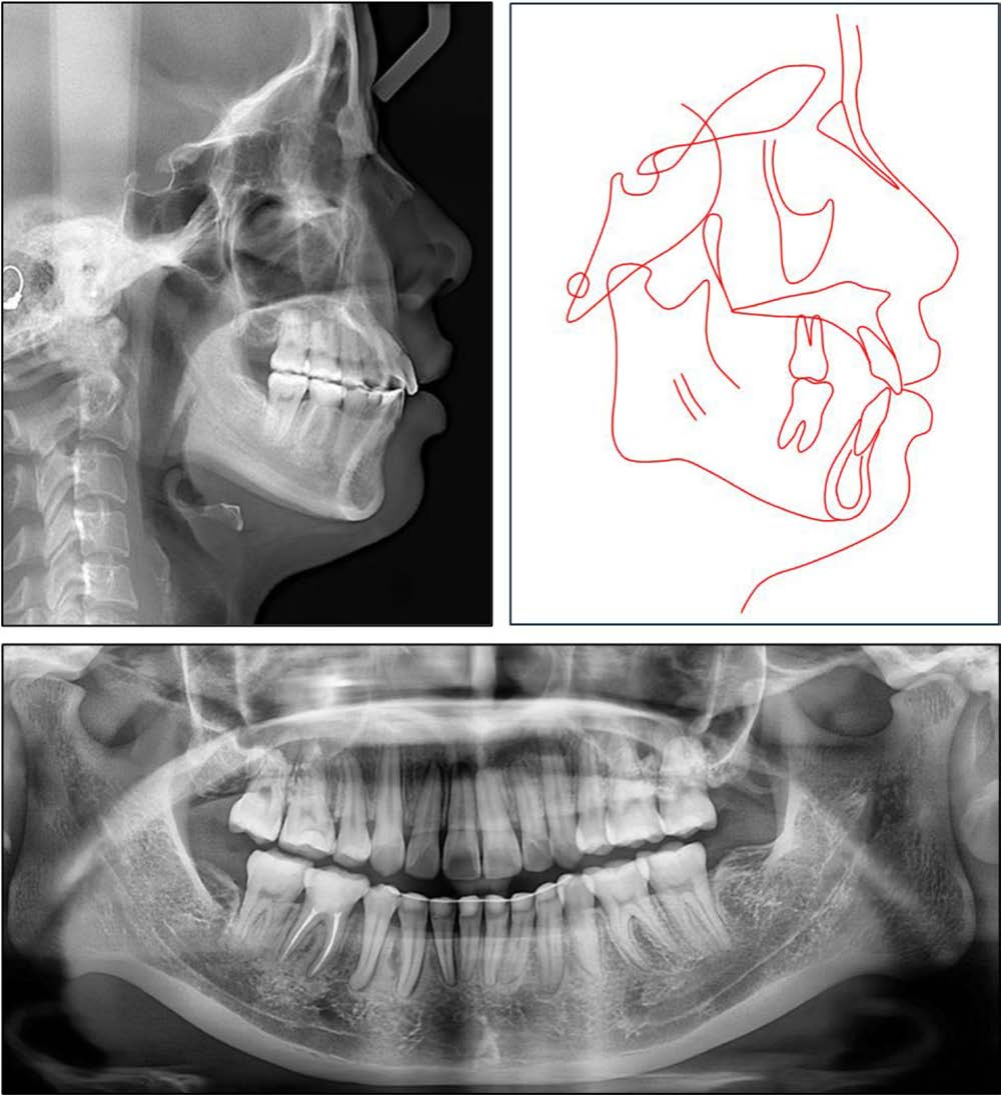
Posttreatment cephalometric and panoramic radiographs and tracing.

Cephalometric analysis revealed a mild reduction in both sella-nasion-point A (77.02°) and sella-nasion-point B (72.30°) angles. The sella-nasion-point A reduction was likely due to alveolar bone remodeling after maxillary incisor retraction, while the sella-nasion-point B change reflected mandibular movement from the intercuspal position to centric relation. The point A-nasion-point B angle also showed a modest increase to 4.72°. The patient’s initial increased overjet and anteroposterior open bite had led to a habitual forward occlusion compared with the centric relation. Subsequent cephalometric analysis indicated that the condyles had moved to a more superior position within the fossa. The lower anterior facial height remained largely unchanged (Frankfort mandibular plane angle, 31.15°). The maxillary incisors demonstrated a good torque control with minimal lingual tipping (upper incisor to sella-nasion, 98.88°), while the mandibular incisor inclination improved, as evidenced by an lower incisor to mandibular plane angle of 97.84°. Both mandibular and maxillary lip protrusion relative to the E-line showed a noticeable reduction, measuring 0.52 and 1.60 mm, respectively (Fig. 6).

**Figure 7.**
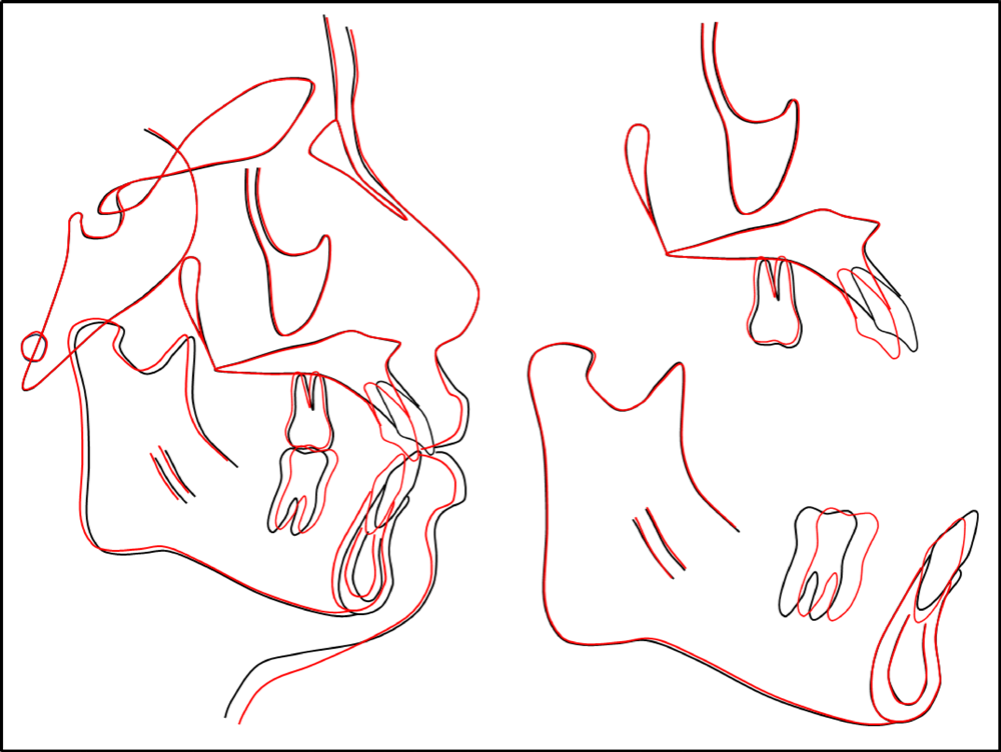
Overall, maxillary, and mandibular cephalometric superimpositions: black, pretreatment; red, posttreatment.

Cephalometric superimpositions demonstrated near-bodily retraction of the maxillary incisors, while the mandibular incisors exhibited intrusion and lingual tipping. Placement of the palatal mini-implants effectively maintained the anteroposterior position of the maxillary molars, while the mandibular molars moved mesially (Fig. 7). Panoramic radiographs confirmed parallel root orientation, adequate bone support, and no root resorption. The teeth are now well-aligned without any overlapping. Bone density in the area of the extracted third molar, and removed cyst has also improved.

**Figure 8.**
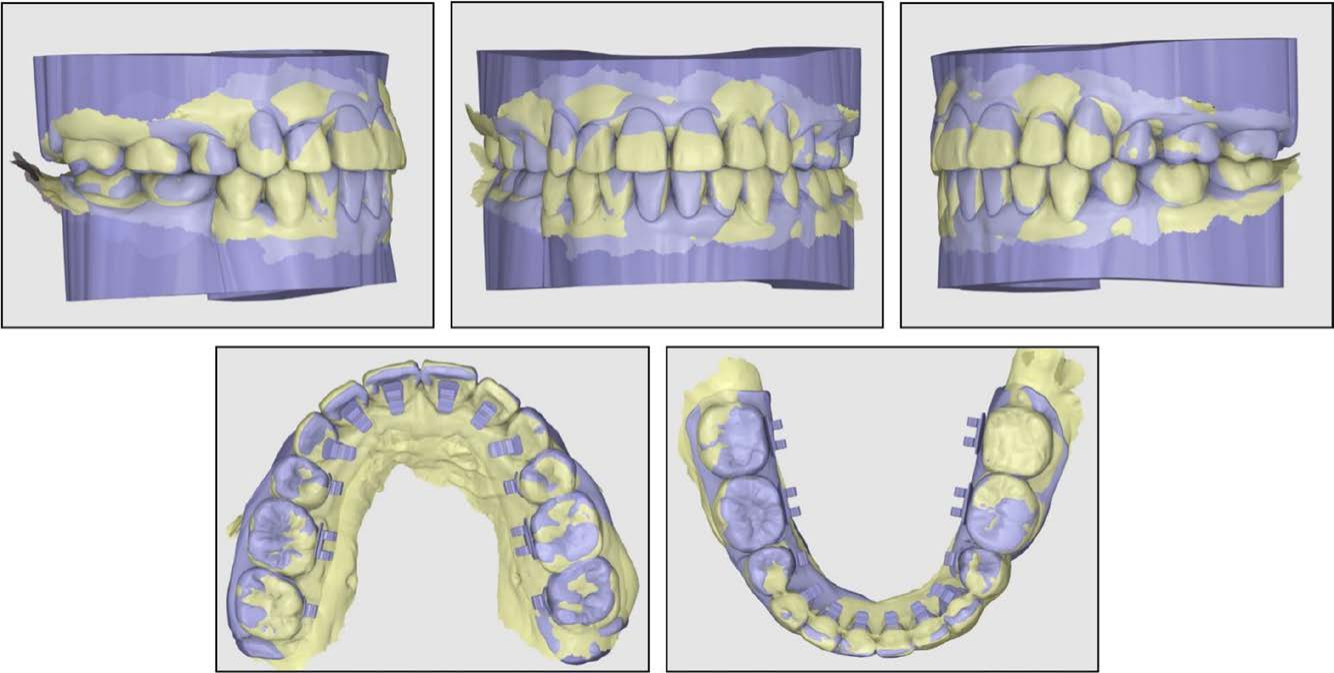
Superimpositions of the posttreatment intraoral scans (yellow) with the orthodontic setup models (blue).

The superimposition of the posttreatment intraoral scan with the planned virtual setup model demonstrated a close correspondence, highlighting the personalization and accuracy of the customized brackets (Fig. 8).

**Figure 9.**
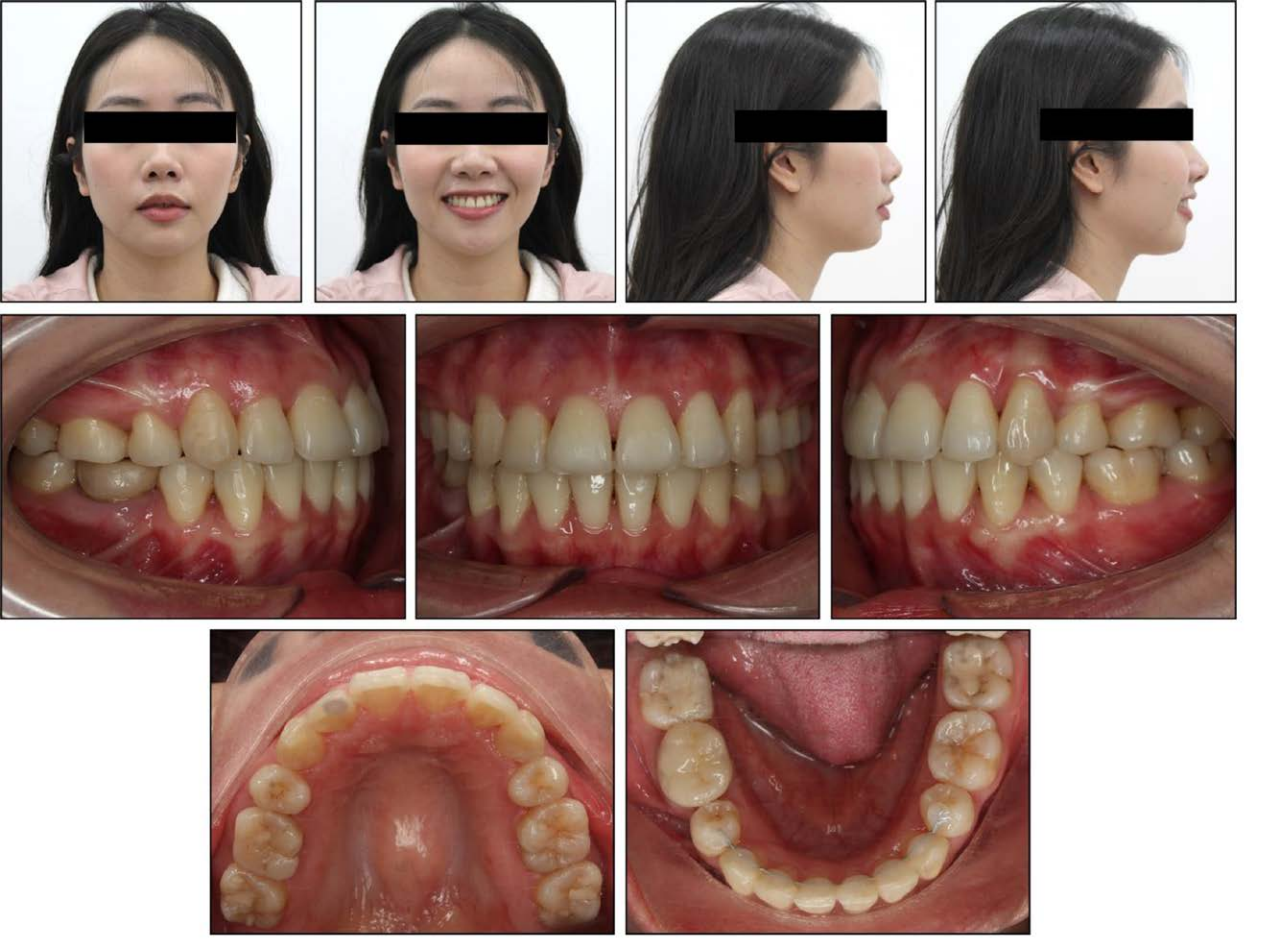
One-year post-retention extraoral and intraoral photographs.

At a 1-year follow-up appointment, the patient exhibited excellent stability of the treatment outcome (Fig. 9). The teeth remained well-aligned, maintaining the achieved Class I occlusion and centered midlines. The patient expressed satisfaction with the long-term results and continued to adhere to the recommended retention protocol.

## 3. Discussion

Utilizing a fully customized 3D-printed lingual appliance system offers orthodontists and dental technicians’ enhanced efficiency and precision. First, the built-in bracket prescriptions are customized for each patient, ensuring optimal final tooth position with proper root placement within the alveolar bone. Second, the base design of these customized lingual appliances is more specialized than regular stock brackets. Its custom-molded shape precisely matches the lingual tooth surface, resulting in a significantly improved fit upon bonding. This enhances bond strength and minimizes the risk of debonding, a particularly valuable advantage for patients in remote areas. Throughout this treatment, only 2 bonding failures occurred, both on the maxillary second molars, possibly due to short clinical crowns and occlusal interferences. This precise fit also allows for greater accuracy when bonding directly without guiding trays, which is particularly useful in cases of bond failures or for bonding brackets on severely displaced teeth that initially hindered bonding.

Beyond company-made systems, several in-clinic or private-lab workflows for customized brackets have been reported. Panayi et al described in-house computer-aided design and 3D printing of customized brackets using hybrid ceramic resins, demonstrating chairside feasibility outside large manufacturers.^[15]^ A related clinical companion detailed in-house customization of lingual brackets with orthodontic computer-aided design, emphasizing practical steps and limitations of small-lab production.^[16]^ Other groups have explored office-based computer-aided manufacturing brackets with composite or ceramic bases printed on benchtop printers, showing acceptable fit and clinical utility when paired with careful bonding protocols.^[17]^ Our workflow expands on these reports by combining open-source base design with laser powder bed fusion metal brackets, leveraging Type-5 Co–Cr properties (high yield strength, hardness, and corrosion resistance) for durability under lingual mechanics.^[10,11]^ Compared with commercial systems, clinic/lab builds provide lower cost and design freedom but demand attention to build orientation and support strategy (e.g., keeping supports off the slot) to preserve slot geometry; these laser powder bed fusion considerations are well documented for dental Co–Cr and were integral to our process.^[12]^ For auxiliary components, recent published in-house printed transfer jigs and indirect bonding trays further streamline adoption in clinics without access to centralized labs.^[18–21]^

Controlling maxillary incisor torque during retraction is a major challenge in Class II malocclusion cases involving premolar extractions. Inadequate built-in torque overcorrection in the bracket prescription can lead to lingual tipping of the maxillary incisors.^[22]^ However, throughout this treatment, changes in maxillary incisor inclination were minimized, and torque was effectively controlled, largely due to the customized brackets. This precise torque control is a significant advantage of customized lingual appliances.^[23,24]^ The high degree of conformity between the planned and achieved occlusion underscores the effectiveness of this customized bracket system in achieving the desired tooth positions, consistent with findings from previous studies.^[7,25]^ The precise control afforded by these brackets facilitated efficient tooth movement and minimized the need for mid-treatment adjustments, such as wire bending.^[7,26]^ This case report demonstrates similar outcomes, further supporting the efficacy of customized lingual brackets in achieving predictable and efficient orthodontic treatment.

In addition to the precision of customized brackets, achieving the planned treatment outcome requires careful coordination of the maxillary and mandibular arches using external mechanics, including strategic extractions, mini-implants, and intermaxillary elastics. Orthodontic treatment combining customized lingual brackets with mini-implants has demonstrated excellent results, even in complex cases. For instance, Bian et al reported a case involving an open bite and crossbite successfully treated with customized lingual appliances, extractions, and mini-implant-supported distalization.^[27]^ Similarly, Inami et al and Wang et al reported successful treatment of Class II high-angle malocclusions and severe Class II malocclusions with excessive overbite and overjet, respectively, using customized lingual appliances and extraction of maxillary first and mandibular second premolars.^[28,29]^

While customized lingual brackets offer numerous advantages, they also have drawbacks. Notably, research by Kyprianou et al has shown that these appliances generate higher initial forces during treatment compared with labial brackets, particularly when combined with rectangular archwires.^[30]^ Their study also found that premolar rotation can be more challenging with customized lingual brackets compared with labial brackets. However, in this case, the patient presented with severe crowding in both arches, necessitating the use of more resilient wires initially to mitigate excessive force.

Second, aside from aesthetic concerns for both patients and doctors, the comfort level during orthodontic treatment is also crucial. A study by Yuan et al compared discomfort and pain levels between buccal and lingual braces, revealing that despite greater speech difficulty and tongue pain with lingual appliances, patients experienced less cheek and lip pain, resulting in a similar overall level of pain, as well as difficulty in chewing and tooth brushing.^[31]^ However, in this particular patient, there was no reported discomfort or tongue injury throughout the treatment, and speech difficulties only persisted for 1 to 2 weeks after lingual bracket placement.

Finally, the high investment and operational costs associated with 3D metal printing systems often limit their direct implementation in private clinics, necessitating the involvement of manufacturing laboratories. However, ongoing advancements in technology are leading to the development of promising portable, small-size metal 3D printers. These advancements hold the potential to revolutionize orthodontic treatment by enabling chairside fabrication of customized appliances, increasing efficiency, and reducing turnaround times.

One limitation of this case report is the use of conventional ligating brackets instead of self-ligating lingual brackets. As reported in several studies, self-ligating customized lingual bracket systems can significantly reduce the chairside time for dental assistants during routine checkups by eliminating the need for steel or elastomeric ligatures.^[2,32]^ However, current metal 3D printing technology has yet to achieve the precision and smoothness required for printing complex self-ligating mechanisms. This limitation may be overcome in the future with further advancements in 3D printing technology, potentially leading to even more efficient and patient-friendly treatment options.

## 4. Conclusion

This case report demonstrates the successful treatment of a Class II malocclusion with significant arch-length discrepancies and midline deviations using 3D-printed customized lingual appliances. By addressing the patient’s chief complaint of crowding and achieving a harmonious occlusion and balanced profile, this approach highlights the potential of digital technologies to enhance orthodontic treatment. Further advancements in 3D printing technology are expected to expand the applications and accessibility of customized lingual appliances.

## Author contributions

**Conceptualization**: Viet Anh Nguyen.

**Data curation**: Viet Anh Nguyen, Thi Minh Anh Ha.

**Investigation**: Viet Anh Nguyen.

**Methodology**: Viet Anh Nguyen.

**Software**: Viet Anh Nguyen.

**Supervision**: Viet Anh Nguyen.

**Writing – original draft**: Viet Anh Nguyen, Thi Minh Anh Ha.

**Writing – review & editing**: Viet Anh Nguyen.
